# Crosstalk and plasticity driving between cancer-associated fibroblasts and tumor microenvironment: significance of breast cancer metastasis

**DOI:** 10.1186/s12967-023-04714-2

**Published:** 2023-11-17

**Authors:** Wenfeng Zhang, Jia Wang, Cun Liu, Ye Li, Changgang Sun, Jibiao Wu, Qibiao Wu

**Affiliations:** 1 State Key Laboratory of Quality Research in Chinese Medicine, and Faculty of Chinese Medicine, Macau University of Science and Technology, Avenida Wai Long, Taipa, 999078 Macau China; 2 College of Traditional Chinese Medicine, Weifang Medical University, Weifang, 261000 China; 3 College of Traditional Chinese Medicine, Shandong University of Traditional Chinese Medicine, Jinan, 250355 China; 4 Department of Oncology, Weifang Traditional Chinese Hospital, Weifang, 261000 China

**Keywords:** Breast cancer, Cancer-associated fibroblasts, Tumor microenvironment, Metastasis

## Abstract

Cancer-associated fibroblasts (CAFs) are the most abundant stromal cell population in breast tumors. A functionally diverse population of CAFs increases the dynamic complexity of the tumor microenvironment (TME). The intertwined network of the TME facilitates the interaction between activated CAFs and breast cancer cells, which can lead to the proliferation and invasion of breast cells. Considering the special transmission function of CAFs, the aim of this review is to summarize and highlight the crosstalk between CAFs and breast cancer cells in the TME as well as the relationship between CAFs and extracellular matrix (ECM), soluble cytokines, and other stromal cells in the metastatic state. The crosstalk between cancer-associated fibroblasts and tumor microenvironment also provides a plastic therapeutic target for breast cancer metastasis. In the course of the study, the inhibitory effects of different natural compounds on targeting CAFs and the advantages of different drug combinations were summarized. CAFs are also widely used in the diagnosis and treatment of breast cancer. The cumulative research on this phenomenon supports the establishment of a targeted immune microenvironment as a possible breakthrough in the prevention of invasive metastasis of breast cancer.

**Graphical Abstract:**

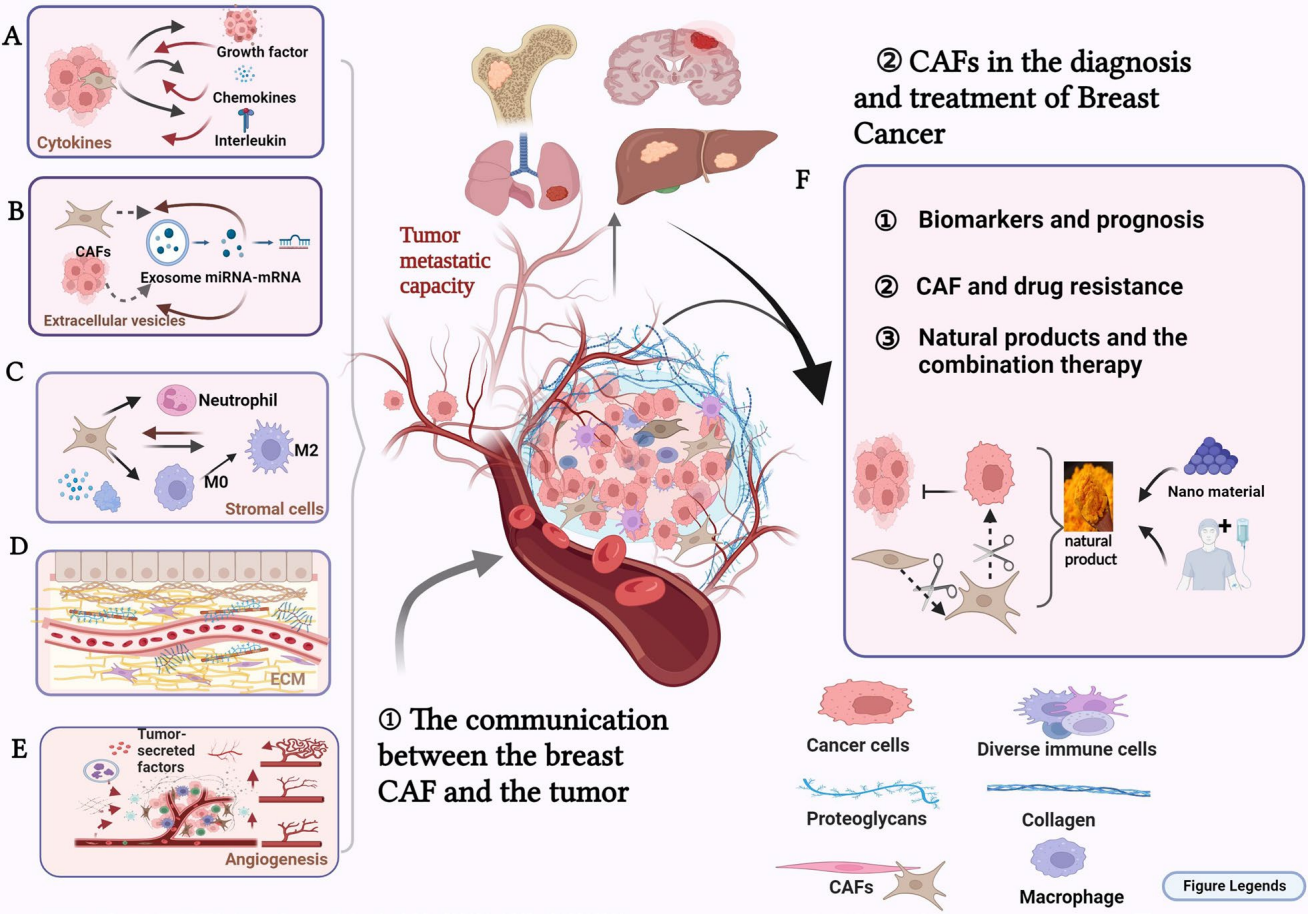

## Introduction

Metastasis of breast cancer is the main cause of cancer-related death among women worldwide [1]. Although breast cancer occurs in breast epithelial cells, an increasing number of studies have confirmed that breast stromal cells also play an important role in tumor metastasis. The heterogenous interaction between cancer cells and stromal cells leads to the proliferation and metastasis of malignant cells [2]. The tumor–matrix ratio has been shown to be an independent prognostic factor in patients with breast cancer. The content of stromal cells and proliferation of matrix connective tissue are significantly correlated with the poor prognosis of breast cancer [3, 4]. Owing to the extensive hyperplasia of connective tissue in the breast, CAFs account for 80% of the tumor mass and are the most common stromal cell component in the breast TME [5, 6]. CAFs and the complexity of the TME affect each other, worsening tumor progression [7]. The transformation and activation of CAFs is one of the basis of cancer progression [8]. When fibroblasts are overactivated, they no longer carry out tissue repair but become harmful components that promote organ fibrosis or tumor growth [9]. CAFs can achieve tumor survival and continuous spread through different mechanisms, mainly through the release of secreted paracrine factors, cytokines, and exocrine vesicles and also through direct or indirect interactions with other cells to achieve physical remodeling of the ECM and ultimately enhance the motility of cancer cells, resulting in internal or distant metastasis [10].

To date, the attempts the programs to reverse or and reshape the TME have mainly focused on cutting off the relationship between different elements in the tumor environment. In this context, we focus on the effects of crosstalk between CAFs and different elements of the TME on factors of tumor metastasis, specifically chemokines, growth factors, immune cells, ECM, and other factors promoting tumor progression, we summarize how fibroblast differentiation and tumor metastasis can be inhibited by regulating various factors in the TME. In addition, the integrated application of natural products and nanomaterials has attracted wide attention as a new approach for targeting the TME or inhibiting tumor metastasis through CAFs [11]. Solving the complex relationship between CAFs and breast cancer metastasis is an important step to break through the bottleneck of clinical treatment of breast cancer at different stages, including advanced breast cancer [12].

## Characteristics of breast CAFs

### Origin of breast fibroblasts

**Fig. 1:**
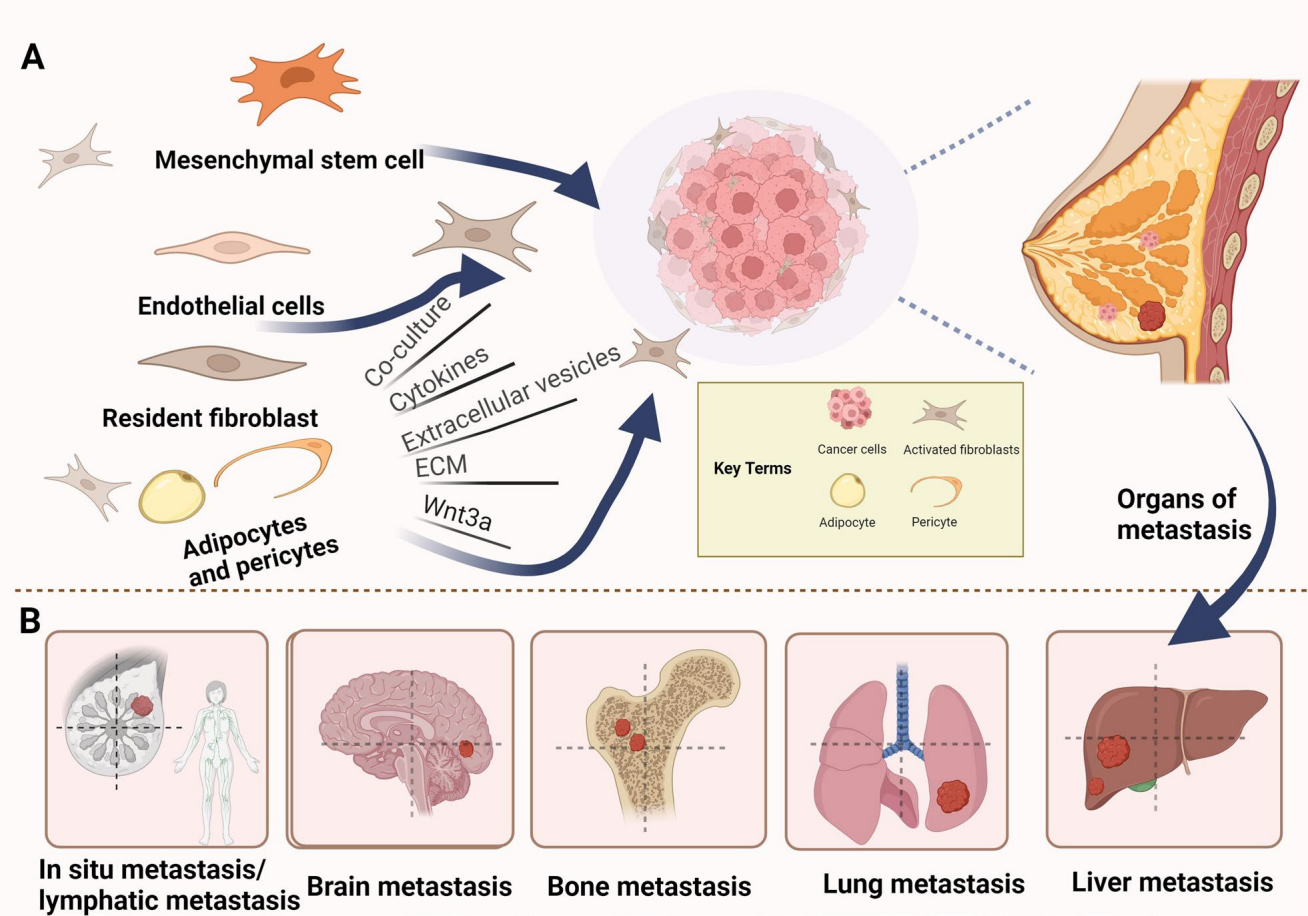
The source of breast cancer-associated fibroblasts and the main organs of breast cancer metastasis. **A** Origin of fibroblasts. **B** Metastatic organs of breast cancer

As a novel cell population, CAFs are extremely heterogeneous and can originate from totally different cell precursors and locations. However, in the context of breast cancer, sources of CAFs are limited. As shown in Fig. 1, we have given a simple indication of the classification of CAF breast cancer. Breast CAFs are typically derived from some main sources: resident fibroblasts, bone marrow-derived mesenchymal stem cells (MSCs), cells that undergo epithelial or endothelial-interstitial transformation, and adipocytes and pericytes [5, 13, 14]. First of all, in breast cancer, most CAFs may originate from the activation of local tissue resident fibroblasts, such as miRNA may be the key mediator of activated CAF-induced cancer metastasis. Down-regulation of miR-200 can induce CAF-like features in normal assciated fibroblasts (NFs) [15]. At the same time, some transcriptional regulators and exocrine can also play this activating role [16];Breast CAFs are also derived from MSCs. Proinflammatory cytokines like tumor necrosis factor-α(TNF-α) and interleukin-1β(IL1β) cause MSCs to transform into inflammatory CAFs [17]. In addition, long-term exposure of MSCs to tumor conditions derived from human breast cancer cells induces myofibroblast-like features in CAFs [18]. Breast CAFs may be derived from epithelial, endothelial, or cells that undergo epithelial or endothelial–mesenchymal transformation; although endothelial cells and epithelial cells are not part of the fibroblast lineage, they can differentiate into CAFs. Bartoschek analyzed different breast CAFs subsets using scRNA-seq, and the transcriptional characteristics and histological localization of some samples suggested that they originated from the epithelium [19]. In addition, fat cells and pericytes are sources of CAFs, and adipose tissue is a rich source of MSCs. Studies have shown that tumors preferentially recruit usable stromal cells from nearby tissues such as fat cells. The production of adipocyte-derived fibroblasts depends on the reactivation of the Wnt/β-catenin pathway in response to Wnt3A secreted by tumor cells [20]. Similarly, the analysis of some breast CAFs subsets of scRNA-seq provides evidence that CAFs originate from pericytes; this diversity of CAFs sources relates to the complexity of the TME [19]. In a sense, the controversy over the most likely origin of CAFs points to the heterogeneity of CAFs sources, which indicates that tumor fibroblasts exhibit multiple functions [21]. With the change in the “soil” of the TME, fibroblasts and their progenitor cells will dynamically change accordingly, which may lead to anti- or pro-tumor activity [22].

### Morphology and activation of fibroblasts

In normal human tissues, resting fibroblasts are identified (under a microscope) as monolayer cells, with a slender spindle shape, that originate from the mesoderm and are fusiform [23]. Under normal conditions, fibroblasts remain in a static and non-proliferative state, and resting CAFs are similar to normal fibroblasts in a steady state. In contrast to static fibroblasts, activated fibroblasts have large fusiform cells, rich basophilic cytoplasm, zigzag nuclei, rich Golgi complexes, and tension fibers visible under an electron microscope [24]. The activation of quiescent fibroblasts into activated fibroblasts was first observed against the background of acute inflammation. When normal fibroblasts are exposed to stimuli, such as persistent inflammation and tumor-related lesions, they reversibly or irreversibly change from a quiescent state to an activated state, and quiescent fibroblasts are activated into myofibroblasts against the background of inflammation. The transformation of stromal cells in TME may be an effective strategy for tumor therapy, so more researchers focus on targeting tumor microenvironment [25].

## Role of CAFs in breast cancer metastasis

**Fig. 2:**
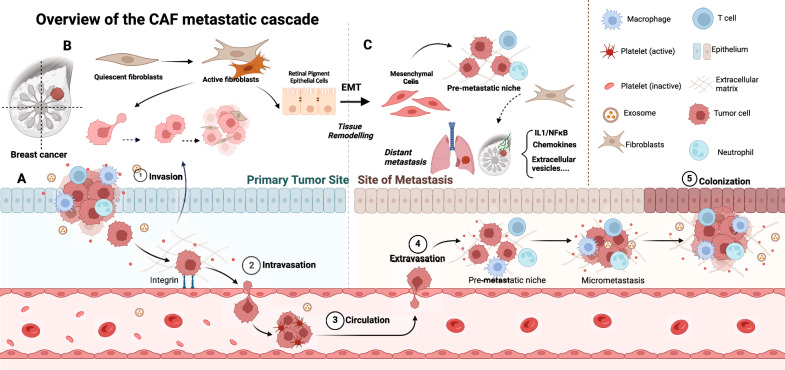
The progression of tumor metastasis. **A** The process of tumor metastasis, which is mainly divided into two parts: primary tumour site and site of metastasis. **B** CAFs and Invasion of breast cancer in situ. **C** CAFs and distant metastasis

CAFs drive breast cancer progression by allowing cancer cells to acquire an aggressive phenotype through the cross-reaction of different factors, which result in the unlimited proliferation of breast tumors, colonization and dissemination of tumors before metastatic spread, and increased permeability of the endothelial cell layer in a specific way that enables tumor cells to escape from the primary site to other distant organs [26, 27]. In terms of tumor progression, tumor metastasis occurs not in a single way, but in multiple stages simultaneously. Tumor-associated fibroblasts not only promote the progression of primary tumor metastasis, but also play an important role in distant metastasis. Fibroblasts carried from primary tumors increase the efficiency of lung metastasis, and the existence and potential direct involvement of fibroblasts in cancer patients [28]. The process of fibroblasts in tumor metastasis is shown in Fig. 2.

### The process of tumor metastasis in situ

Tumor metastasis includes a series of biological events, and the gradual acquisition of the ability of primary tumor cells to invade deeper tissues through the mucosa is considered to be the first step in the beginning of tumor metastasis [29]. When the tumor begins to metastasize, the primary problem is the transformation from single tumor cells to multiple tumor cells. in this process, the core of migration and development is a strong cytoskeleton, which can cope with the pressure related to migration and avoid cancer cell death. In the initial stage, ECM plays an important role as a key factor in the composition of the structure. The detachment of cells from the primary tumor site involves epithelial–mesenchymal transition (EMT). CAF-induced EMT in tumors is the result of multiple factors and conditions [10, 30].

### Distant metastasis of tumor

Breast CAFs and cancer cells accumulate and then circulate in the peripheral blood of patients with metastatic breast cancer to form a niche for distant organ metastasis, ultimately completing the metastatic cascade. Breast cancer fibroblasts play an important role in the transmission of the pro-cascade, whether in the infinite proliferation of cells, intravasation of cells, or formation of metastatic niches. There is also a communication mechanism between primary tumor and distant metastasis. Metastatic cancer cells usually exist in dormant state in distal tissues and organs [31]. As the driver of pre-metastatic niche, breast cancer -associated fibroblasts play a role in the colonization of metastatic organs. Angiogenesis, extracellular vesicles and cytokines are closely related to the pretumor niche in this process. CXCL12 is the main driver of vascular permeability, which promotes the infiltration of tumor cells and enables tumor cells to escape from the primary site to other distant organs [32, 33]. Interleukin-induced chemokine is also another driving point of tumor metastasis. IL-1 ɑ and IL-1 secreted by breast cancer cells induce lung fibroblasts to produce CXCL9 and CXCL10 through NF- ĸ B signal, thus promoting the growth of lung metastases [34, 35]. Cav-1 in exocrine bodies derived from Breast cancer (BC) can be used as signal molecules to promote the secretion of tenascin-C in lung fibroblasts and lead to the deposition of ECM, which mediates intercellular communication and regulates premetastatic niche (PMN) before lung metastasis [36]. lncSNHG5 in CAFs and its downstream signal ZNF281-CCL2/CCL5 are associated with pre-metastatic niche formation in breast cancer, in which CAFs-regulated ZNF281 has a regulatory relationship with angiogenesis and vascular leakage [37]. In addition, lymph nodes are also an important involved organ in the pre-tumor niche, with fibroblast subsets CAF-S1 and CAF-S4 accumulating in metastatic lymph nodes and associated with cancer cell invasion [38]. There are few studies on how cancer cells use CAFs microenvironment to establish pre-metastasis microenvironment. At present, it mainly involves lung colonization metastasis in distant metastasis of breast cancer, while distant metastasis in other organs is still blank. Breast cancer metastasis mediated by CAFs is a promising development direction.

In this process, CAFs secrete a variety of cytokines, such as growth factors and chemokines, which regulate the TME and adapt it to sustain tumor growth; CAFs also recruit immune cells that change the immune microenvironment. In addition, CAFs increase the deposition and remodeling of ECM, increase the tension and stiffness of cells and tissues, and change the tissue structure [39, 40]. CAFs-mediated reactions mainly occur through cross-reactions with multiple levels of primary cancer cells and other components of the, leading to the uncontrollable development of tumors. This uncontrollable development is not only manifested in the crosstalk between cytokines, immune cells, extracellular vesicles, and other single factors but also involves a complex cascade of signaling pathways [41].

## CAFs under the complex interweaving of tumor metastasis microenvironment

**Fig. 3:**
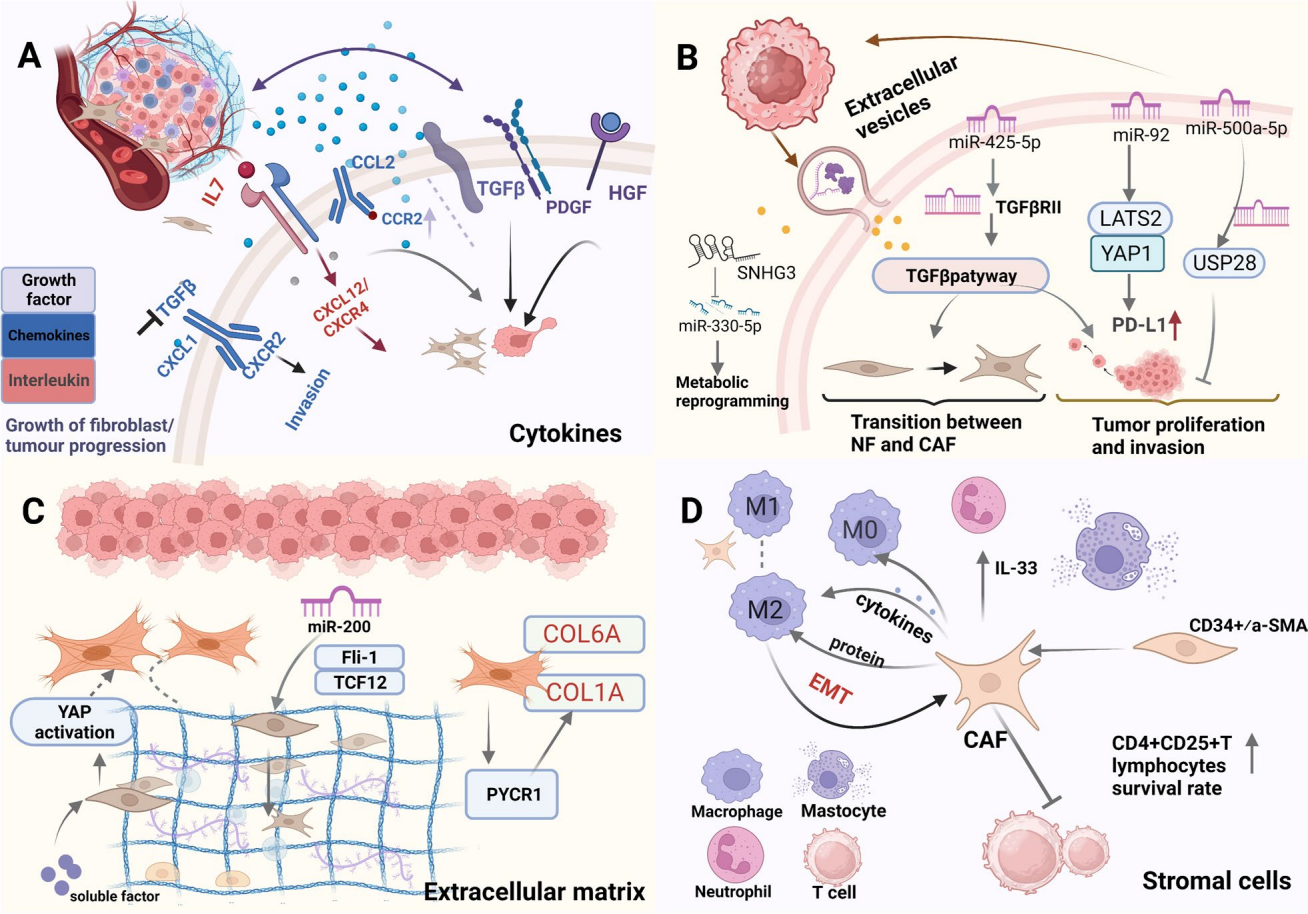
Communication between breast CAFs and tumor. CAFs regulates the growth and invasion of cancer cells by secreting cytokines, exosomes, ECM and stromal cells. **A** The mode of action of cytokines (growth factors, chemokines, interleukins)** B** CAFs cut off the transfer pathway by targeting exosomes **C** Remodeling of the extracellular matrix **D** Relationship between CAFs and stromal cells (mainly macrophages)

The effects of activated CAFs on tumors are multifaceted and include tumor colonization, metastasis, and unlimited dissemination [42, 43]. An important step in the transformation of the precancerous microenvironment is the transformation of normal interstitial fibroblasts into CAFs [8]. Consuming active CAFs or reprogramming active CAFs into static fibroblasts is the first strategy to block stroma formation in breast cancer. This blocking can be achieved by directly targeting the formation of CAFs and indirectly eliminating the formation pathway of CAFs. Various factors in the TME communicate with each other to promote the progression of tumor metastasis, and cutting off the communication link between the tumor and the microenvironment can also block the progression of tumor metastasis. The main influencing factors in CAFs and TME are shown in Fig. 3.

### Cytokines secreted by CAFs: CAFs communication with tumor metastasis

CAFs secrete various cytokines including growth factors, chemokines, and interleukin. These factors not only act as important stimulators to directly activate CAFs but also target key signal axes to promote tumor progression [44]. Among the large number of crosstalk factors, cytokines have attracted the attention of researchers The pathways of action of various cytokines are described in Table 1. In a sense, CAFs act as a “regulatory center” in the TME. The relationship between CAFs and various chemotactic factors can be regulated dynamically and bidirectionally, similar to a bidirectional switch. CAFs are mainly involved in three types of cytokines, growth factors, chemokines, and interleukins, in the TME.

**Table 1:** Regulatory relationship between different types of cytokines and CAFs in the TME

Type of factor	Molecules	Secretory cell	Underlying mechanisms	Refs.
Growth factor	TGF -β	CAFs	CAFs secretes a high level of TGFβ and CAFs activates HOTAIR transcription through TGF β-1 secretion, thus promoting the metastatic activity of breast cancer cells	[48]
	TGF -β	CAFs	TGFβ and inflammatory cytokines secreted by breast cancer cells stimulated GREM1 expression in CAFs. Grem1 disrupts bone morphogenetic protein (BMP)/SMAD signaling in breast cancer cells, promoting their mesenchymal phenotype and invasiveness	[50]
	TGF-β/SMAD	CAFs	CAFs promote an aggressive phenotype of breast cancer cells through paracrine TGFβ-induced EMT	[49]
	TGF-β	CAFs	Knockdown FAP-α leads to EMT reversal and elimination of TGF-β1-activated CAF-induced tumor invasion and lung metastasis	[70]
	TGF-β	CAFs	ECM hardness in TGF-β-related pathways can build bridges across the basement membrane, and CAFs are major contributors to ECM stiffness and degeneration, both of which contribute to cancer cell invasion	[71]
	CTGF/TGF-β	CAFs	Nicotine-treated fibroblasts producing CTGF and TGF-β have proven essential for promoting EMT and cancer cell migration, as well as blocking CTGF and TGF-β in tumor motility by blockingNic-CM inhibition of tumor motility	[72]
	TGF-β	CAFs	Loss of Tgfbr2 expression in breast fibroblasts is associated with tumorigenesis and metastasis	[73]
	TGF-β	CAFs	CAFs promote aggressive phenotyping of breast cancer cells through paracrine TGF-β1-induced EMT	[49]
	HGF	CAFs	HGF secreted by fibroblasts has been shown to mediate proliferation and invasion of cancer cells	[51]
	FGF2	CAFs	CAFs promote the growth, migration and invasion of MDA-MB-231 cells through paracrine FGF2-FGFR1 loop	[52]
	IGFs	BCs	The transformation of breast epithelial cells and stromal fibroblasts to CAFs is connected by IGFs/IGF-1R axis, which directly promotes TME remodeling and increases tumor invasion	[53]
	TGF-β, PDGF	CAFs,Breast cancers (BCs)	TGF-β and platelet derived growth factor (PDGF) play a clear role in promoting tumor phenotype of fibroblasts	[54]
Chemokines	CCL2/CCR2	CAFs	The overexpression of CCR2 in ductal carcinoma enhances the invasive progress associated with fibroblast accumulation	[61]
	CXCL12	CAFs	CAFs regulates the mdia2-directed cytoskeleton in breast tumor cells by secreting CXCL12, thus promoting the mechanism of tumor cell migration and invasion	[62]
	CXCL12	CAFs	OPN produced by cancer cells and CXCL12 secreted by activated fibroblasts trigger EMT in breast cancer	[63]
	CXCL12	CAFs	CXCL12 secreted by fibroblasts plays an important role in promoting angiogenesis and tumor cell infiltration	[26]
	CXCL1/CXCR12	CAFs	phenotype of BCAHC-4 cells triggered by paracrine connections between insulin-activated tumor cells and CAFs on the CXCL1/CXCR12 axis	[64]
	CXCL8	BCs	Tumor-stromal interactions provide the basis for activation of Notch1 leading to CXCL8 secretion and consequent pro-metastatic activity	[65]
	CCL2,CCL5,CXC-related chemokines	CAFs	These factors released by the inflammatory CAFs enhance the dispersal and migration of tumor cells	[17]
Interleukin	IL-6	CAFs	Elevated IL-6 in supernatants of isolated CAFs suppresses HIC1 expression in cancer cells and promotes breast cancer development in the TME through paracrine or autocrine signaling	[66]
	IL-6	BCs	miR-216a regulates CAFs crosstalk in cancer cells by modulating the TLR4/IL6 pathway	[67]
	IL-11	BCs	Interleukins secreted by BC promote migratory and invasive characteristics of mammary CAFs	[69]
	IL32	CAFs	CAFs-secreted IL32 promotes breast cancer cell invasion and metastasis through integrin β3-p38MAPK signaling	[68]

#### Growth factors

Some growth factors not only activate fibroblasts directly as stimulating factors but also regulate the environment by secreting a large number of autocrine and paracrine cytokines and other tumor-promoting factors by CAFs to build an environment conducive to tumor growth and transplantation [45, 46].

The most representative growth factor is transforming growth factor-β (TGF-β), which is often involved in ECM mechanical sensing and myofibroblast differentiation [47]. Studies have found that cancer-related fibroblasts secrete more TGF-β and activate the TGF-β/Smad signaling pathway in breast cancer cells. Tumor cells can secrete both TGF-β and paracrine TGF-β. The transformation of interstitial fibroblasts into CAFs is mediated by the paracrine action of tumor cells. Moreover, studies have confirmed that cancer-related fibroblasts induce tumor progression by secreting TGF-β1, involving all stages of tumor invasion [48, 49]. The secretory growth factor Bone morphogenetic proteins of the TGF-β family was co-injected into a xeno-transplanted zebrafish model; Grem1, a Bone morphogenetic proteins antagonist produced by CAFs, was found to promote the activation of fibroblasts and infiltration and extravasation of breast cancer cells, thus, promoting the formation of Micrometastases. This physical dissemination is the first step in the invasion–metastasis cascade reaction [50]. Subsequently, tumor progression requires continuous adaptation and modification of the metastatic microenvironment. It has been found that CAFs promote the invasive phenotype of breast cancer cells by paracrine TGF-β1-induced EMT [49]. In addition, CAV-1 deficiency in fibroblasts increases the secretion of TGF-β, which in turn activates the TGF-β/Smad signal pathway in breast cancer, thus, promoting the metastasis and dryness of breast cancer tumors.

In addition to the typical TGF-β pathway, other growth factors play an important role in metastasis. Researchers have found that hepatocyte growth factor (HGF) secreted by CAFs may be one of the contributing factors to the tumorigenic difference between CAFs and NFs. Breast cancer cells reprogram the surrounding fibroblasts to secrete HGF, which is positively related to the enhancement of breast tumorigenesis, migration, and invasion [51]. In addition, CAFs promote the growth, migration, and invasion of MDA-MB-231 cells through the paracrine FGF2-FGFR1 loop [52]. Not only do CAFs secrete HGF, CTGF, basic fibroblast growth factor (bFGF) and other cytokines, which promote tumor metastasis, but the corresponding tumor cells or other types of cell-secreted cytokines can also promote the activation of CAFs to stimulate TME remodeling and increase tumor invasion. For example, the transformation of breast epithelial cells and stromal fibroblasts to CAFs is connected by the IGF/IGF-1R axis, which not only changes the environment but also promotes the progression of the tumor [53]. These effects are obviously bidirectional: the interactions between tumor cells and fibroblasts constitute a vicious cycle of tumor progression. For example, Platelet derived growth factor (PDGF) and TGF-β produced by tumor cells were shown to induce fibroblasts activation, and activated fibroblasts secrete IL-6 to promote tumor cell proliferation and chemotherapy resistance [54].

Nerve growth factor(NGF) is also an important growth factor in other growth factors. CAFs has been shown to activate YAP1/TEAD1 signal and increase the score of NGF in prostate cancer [55]. Moreover, studies have confirmed that ProNGF, NGF and its receptors also play a role in breast cancer proliferation, ECM remodeling, angiogenesis, invasion and metastasis, and there is a correlation between the existence of nerve fibers and the expression of NGF in cancer cells [56]. NGF and its receptor may represent a good diagnostic and prognostic tool and a promising therapeutic target for breast cancer [57–59]. However, no related research has focused on breast cancer metastasis and fibroblast in this field, and it also gives us a positive hint in the research direction.

#### Chemokines

Chemokines and their receptors mediate chemotaxis, which is strongly involved in the dynamic processes of tumor development and progression. Chemokines, as a key factor in the secretion of CAFs, become the “booster” of breast cancer progression [60]. Chemokines are closely related to not only the ability of cancer cells to acquire an aggressive phenotype but also enhanced colonization before the metastatic spread of tumors [27]. Signaling by CCL2/CCR2 is also a transmission pathway of chemokines regulated by CAFs. CCR2 overexpression in ductal carcinoma in situ is known to enhance aggressive progression associated with the accumulation of CCL2-expressing fibroblasts [61].

CAFs and chemokine CXCL12 are the most often studied in the invasive progression of breast cancer. CAF-derived chemokines secrete CXCL12 to regulate the mdia2-directed cytoskeleton in breast tumor cells to affect the movement of breast tumor cells [62]. In addition, CXCL12, cancer cells secrete the chemokine-like protein osteopontin (OPN), and OPN-driven CAFs then secrete CXCL12, which in turn triggers modification of the EMT in tumor cells [63]. CXCL12 derived from CAFs can not only regulate the TME by targeting endothelial cells to promote breast cancer metastasis but also increase the permeability of the endothelial cell layer in a specific way. CXCL12 is a driving factor for tumor cell and enhances vascular permeability, which enables tumor cells to escape from the primary site to other distant organ [26]. CXCL12 also plays a primary role in metabolism. After the occurrence of metabolic disorders, insulin activates the paracrine connection between tumor cells on the CXCL1/CXCR12 axis and CAFs, triggering the movement phenotype of tumor cells [64].

The stimulation of inflammatory factors by chemokines and the TME cannot be ignored. When tumor cells interact with stromal cells in the presence of pro-inflammatory stimuli, this interaction lays the foundation for pro-inflammatory signals to activate Notch1, resulting in an increase in CXCL8 production and increased metastatic activity [65]. Unlike tumor cells interacting with stromal cells during pro-inflammatory stimulation, pro-inflammatory cytokines can transform MSCs into CAFs. Inflammatory CAF-derived factors promote cancer cell migration by stimulating the chemokines CCR2, CCR5, and CXCR1/2 expressed by cancer cells and Ras-activating receptors [17]. There is no denying the role of inflammatory factors in the microenvironment of breast cancer. Inflammatory factors can transform ordinary cells into tumor-related fibroblasts; the concomitant increase in chemokines provides conditions for the continuous spread of cancer cells.

#### Interleukin(IL)

In addition to chemokines and growth factors, interleukin contribute to the crosstalk in the TME. We investigated the interactions between interleukin and fibroblasts in breast cancer. Interleukin-6 (IL-6) is the most widely studied cytokine. It is undeniable that IL-6 is usually secreted at high levels in the breast cancer microenvironment. Isolation of stromal fibroblasts from breast cancer tissue and analysis of the supernatant showed that IL-6 downregulated the tumor suppressor HIC1 and promoted the development of breast cancer in the TME through paracrine or autocrine signals [66]. In addition to microRNA, miR-216a regulates the crosstalk between cancer cells and CAFs by regulating the TLR4/IL6 pathway. In the face of such an influence, researchers have found that the IL-6 receptor inhibitor tocilizumab effectively and sustainably inhibited the expression of various CAF biomarkers, inhibiting tumor growth while also inhibiting the ability of CAFs to promote EMT. On this basis, inhibiting the angiogenesis promotion of activated CAFs in vitro and in situ (tumor xenografts) has become a new avenue for CAFs targeted therapy [67]. Other related interleukins still have great potential to support targeted therapy for CAFs. For example, the IL32 protein secreted by CAFs binds to integrin β3 on the surface of breast cancer cells, thereby activating the downstream p38MAPK pathway and enhancing the invasion and metastasis of breast cancer cells [68]. IL-11 regulates the “ECM tissue” signal pathway of tumor-promoting genes, and the related inhibitors and blocking pathways have become a new way to inhibit the formation of tumor metastasis [69].

### Exosomes secreted by CAFs: transport pathway for tumor metastasis

Exosomes are important communication media between tumors and CAFs that encapsulate a variety of proteins, lipids, mRNA, microRNAs, and lncRNAs. Crosstalk between various tumor cells is an important factor that cannot be ignored as a factor in tumor progression. Crosstalk between tumor cells and the microenvironment is not unidirectional. MicroRNAs not only regulate the relationship between NFs and CAFs, but exosomes secreted by tumor cells also play a role in regulating the upstream of CAFs [74]. In turn, the exosomes secreted by CAFs may be internally utilized by tumor cells to promote cancer progression. miRNAs are the main RNA components of extracellular vesicles, and CAFs carrying miRNAs mediate the progression and metastasis of breast cancer. Here, we focus on the relationship between miRNAs and tumor fibroblasts. Most miRNAs are tumor-promoting factors, such as miR-425-5p, miR-146a, and miR-500a-5p, which promote the proliferation, invasion, and migration of CAFs and stimulate tumor growth in vivo [74, 75]. Scognamiglio found that triple-negative breast cancer (TNBC) cell-derived exosomes, miR-185-5p, miR-652-5p, and miR-1246, can synergistically activate stromal fibroblasts to transform into specific CAFs subtypes and promote TNBC cell metastasis and invasion. This modulation of crosstalk also provides an adjuvant therapeutic target for the treatment of breast cancer [30].

Exosome miRNAs promote tumor metastasis and progression by delivery to recipient cells to regulate the expression of target genes. Patient-derived exosome miR500a-5p can endow breast cancer cells with an invasive phenotype [76], and CAFs exosome-derived miRNAs can be absorbed by adjacent or distant tumor cells. Tumor signal transduction is induced by inhibition of the target mRNA of receptor cells to achieve the transfer mechanism. Breast cancer-derived exosomes induce CAFs to activate the Wnt pathway through the miR-146a–TXNIP axis, thereby enhancing invasion and metastasis of breast cancer cells [75]. CAF-derived exosomes containing miR-181d-5p can promote EMT modification in breast cancer cells by regulating CDX2 and HOXA5 [77]. miR-3613-3p is highly abundant in CAFs exosomes, is transferred to breast cancer cells, and promotes cell growth and metastasis by downregulating SOCS2 expression [78]. MicroRNAs in extracellular vesicles maintain the occurrence and development of cancer and mediate the crosstalk between cancer cells and the TME. We found that in the process of exocrine promotion of tumor progression, the exosomes released by tumor cells also have an effect on CAFs, but in the field of breast cancer, research has mainly focused on the effect of CAFs exosomes on tumors. In this process, it mainly targets the miRNA–mRNA axis to play a role in transfer. In addition, based on their tissue- and development-specific expression, miRNAs can be used as early diagnostic and prognostic markers for tumors. As shown in Table 2, we can see the impact of exosome-derived miRNAs released by tumor cells on CAFs.

**Table 2:** Effect of exosome-derived miRNA released by tumor cells on CAFs

miRNAs (expression)	Effects on cancer cells and mechanism of action	Potential targeting therapy	Refs.
miR-500a-5p	CAFs promotes breast cancer progression and metastasis through exosome miR-500a-5p	By binding to ubiquitin-specific peptidase 28	[76]
miR-92	miR-92 secreted by CAFs significantly promotes tumor progression and impairs the function of tumor-infiltrating immune cells in vivo	LATS2 is thought to be a target gene for miR-92, and LATS2 interacts with YAP1	[79]
miR-185-5p,miR-652-5p,miR-1246	The synergistic effects of miR-185-5p, miR-652-5p, and miR-1246 promote fibroblast migration and contraction	Activate NFs to CAFs	[30]
miR-425-5p	R-425-5p promotes conversion to the CAFs phenotype and increases cell motility	By inhibiting its target gene TGFβRII	[80]
miR-222	MiR-222 overexpression or LBR knockdown is sufficient to induce NFs to exhibit CAFs signatures that enhance migration, invasion, and aging	Works by targeting LBR	[81]
miR-21, miR-143, miR-378	miRNAs exhibit significantly increased ability to form mammary globules, increased stem cells and EMT markers, and promote cell growth	Cell phenotypic changes	[82]
miR-146a	The miR-146a/TXNIP axis activates CAFs and activates the Wnt pathway, thereby promoting the invasion and metastasis of BC cells	Through the miR-146a/TXNIP axis, the wnt path is activated	[75]
miR-16, miR-148a	FAK ablation in CAFs increases the level of exosome miR-148a, thereby reducing the expression of WNT1 and WNT10B in recipient cancer cells	FAK signal transduction pathway	[83]
miR-330-5p	SNHG3 knockdown in CAF-secreted exosomes inhibits glycolytic metabolism by increasing miR-330-5p and decreasing PKM expression in tumor cells	Targeting lncRNA SNHG3	[84]
miR-1-3p	The elevation of miR-1-3p in breast cancer cells inhibits cell viability, invasion, migration and transformation from epithelium to mesenchymal, and inhibits tumor formation and metastasis	Targeting Gli-similar1 (GLIS1)	[85]

### Crosstalk between CAFs and other stromal cells: promotion of tumor metastasis

#### Macrophages

Tumor-associated macrophages (TAMs), derived from circulating monocyte precursors, are the most abundant type of innate immune cells in the immune microenvironment. CAFs and TAMs are active participants in tumor progression and metastasis and play a synergistic role in the TME. Investigators have found that in the vicinity of the accumulation area of CAFs, there was a high infiltration of TAMs around the tumor nest in TNBC [86] which provides the basis for the plasticity of macrophages and fibroblasts for tumor treatment strategies [87]. Tumor cells cultured with CAFs showed increased adhesion to monocytes. CAFs and breast cancer cells are known to effectively recruit monocytes. In addition to inducing direct differentiation into the M2 phenotype, different phenotypic characteristics of the macrophages were observed. CAF-induced macrophages show a special M2 polarization phenotype induced by tumors [88, 89]. M2 macrophages can affect the mesenchymal–mesenchymal transformation of fibroblasts [88].

In most studies of the synergistic effect of CAFs and TAMs, researchers use the co-culture incubation mode, in which cytokines, such as chemokines, can regulate the phenotype of other stromal cells, and the cytokines secreted by CAFs are more often used as active triggers to regulate the phenotype of macrophages. CAF-derived IL-33 significantly enhances TAM recruitment and induces M2 phenotype activation. At the molecular level, the IL-33-ST2-NF-κB-MMP9-laminin signaling pathway mediates cross-communication among various cellular components involved in cancer metastasis [90]. CAF-derived IL-33 significantly enhances TAM recruitment and induces M2 phenotype activation. At the molecular level, IL-33-ST2-NF-κB-MMP9-laminin signaling mediates cross-communication among various cellular components involved in cancer metastasis [91].

Paracrine regulation also occurs between CAFs, TAMs, and tumor cells. OPN secreted by TAMs enhances the secretion of OPN by CAFs, thus, increasing the malignancy of cancer cells. OPN is the key molecule in the tumor–CAF–TAM interaction [92]. A correlation between grades of TAMs and CAFs has also been confirmed in breast cancer. In a model of TNBC cells co-cultured with fibroblasts and macrophages, fibroblasts and macrophages were found to be induced to secrete high levels of IL-8, and the IL-8-CXCR2 axis was shown to be important in regulating the progression of breast cancer [93]. The precise interaction among tumor cells, CAFs, and M2 macrophages enhances the motility of tumor cells. In TNBC, crosstalk between cancer cells and fibroblasts or macrophages enhances the migration and proliferation of cancer cells. This provides a good dynamic basis for the metastasis of tumor cells [93]. Clinical samples also provide evidence of this effect. In a retrospective study of 36 patients with breast tumors, researchers analyzed the morphological diversity of tumors and the clinicopathological parameters of the disease. They found that CAFs were associated with TAM infiltration in patients with TNBC, mainly in the number of macrophages, and the number of SMA+FAP+fibroblasts around the trabeculae was found to be related to lymph node metastasis of breast cancer. It has also been confirmed that the diversity of morphological structures in breast tumors is associated with monocyte recruitment as well as the regulation of macrophage and fibroblast phenotype-related cells [93]. The results of these studies demonstrate the cycle that exists between CAFs, M2-polarized macrophages, and tumors, in which the cross interaction between stromal cells, cancer cells, and secreted cytokines actively promotes the invasiveness of tumor cells and ultimately promotes the escape of cancer cells from primary tumors. The synergistic effect of CAFs should also be considered in treatment strategies in which the focus is altering TAM polarization or reshaping M2 macrophages.

#### *T*umor-associated neutrophils

In many steps of tumor progression and metastasis, we observed that TAMs and tumor-associated neutrophils (TANs) are similar, and TANs are abundant immune cells in the TME. Both TAMs and TANs can regulate antitumor immunity and have a polarization effect [94]. Fibroblasts are the main source of IL-33 in the lung metastasis of breast cancer. IL-33 plays an important role in eosinophil recruitment in vivo. Through transfer load analysis, the inhibition of IL-33 was found to play an important role in reducing eosinophil infiltration [34]. Although TANs play a regulatory role, research on TANs is limited, suggesting that this may be a promising field.

Malignant cells crosstalk with a variety of tumor stroma in the TME, including CAFs, TAMs, and other immune cells, to support their growth and metastasis. CAFs cause various forms of secretion by activating various signaling pathways. These secretions provide a medium for driving immune escape in tumors. Cytokines secreted by CAFs actively trigger factors that regulate macrophages or paracrine regulatory links between CAFs, TAMs, and tumor cells to increase cell migration. These findings suggest that eliminating the crosstalk between CAFs and other stromal cells may be a potential therapeutic strategy for blocking tumor immune escape.

### CAFs activates remodeling ECM to promote tumor invasion

ECM remodeling is not only a necessary step in tumor invasion and metastasis, but also the result of comprehensive effects. The ECM is a complex mixture of structural proteins, proteoglycans, and glycoproteins. The mechanical properties of the ECM are closely related to the role of CAFs. Dynamic remodeling of the ECM leads to changes in tumor cell density, hardness, or tissue changes. Changes in the structure of the TME triggered by mechano-transduction promote the directional migration and invasion of cancer cells, and the biomechanical and physico-mechanical properties unique to the ECM may be important factors in the formation of the metastatic niche and cancer progression [95]. The hardness of the matrix increases in solid tumors in breast cancer [96]. CAFs play an important role in this process. CAFs deposit ECM proteins, secrete growth factors, and contract and remodel the ECM, and there is a regulatory relationship between CAFs activation and ECM. Breast cancer progression is accompanied by elevated mechano-signaling and increased tissue birefringence, implying that ECM hardness promotes malignancy and enhances tumor aggressiveness in patients with breast cancer [97, 98].

#### Interaction and adhesion between cells

The first step in invasion and metastasis is the adhesion and interaction between ECM cells. The ECM affects breast tumor growth and metastasis through signaling pathways, key proteins, and related enzymes. CAFs produce a large number of ECM structural components related to tumor-promoting activities; for example, collagen XII is mainly secreted by CAFs and regulates collagen I fibrillar tissue to promote cancer cell invasion and breast cancer metastasis [99]. In addition, ECM arrangement and density can accelerate the progression of breast cancer by promoting T-cell activation induced by fibroblasts [100].

Furthermore, matrix modification and mechanical transduction regulate various signaling pathways, of which the YAP/TAZ pathway is an important connection between breast CAFs and matrix remodeling. CAF-driven YAP-dependent matrix sclerosis may lead to the proximity of some cancer cells to the rigid matrix. In the co-culture model, CAFs were the most effective in promoting the invasion of breast cancer cells. Moreover, their ability to promote matrix remodeling and invasion, which can regulate the contractile actomyosin cytoskeleton and reduce matrix remodeling of CAFs by blocking MYL9/MLC function, increases with an increase in the stage of the disease [96]. In addition, the MRTF-SRF signal pathway is involved. Compared with ordinary fibroblasts, breast CAFs contain nuclear MRTF, and the expression of many MRTF-SRF target genes increases accordingly. An increase in MRTF-SRF target genes is necessary for contractility and invasion of CAFs. The contraction- and invasion-promoting phenotypes of CAFs reflect the activation of the MRTF-SRF signal and YAP-TEAD signal [101].

#### Key enzymes and proteins for ECM hydrolysis

Tumor cells first come in contact with receptors on the surface of the basement membrane and then secrete degrading enzymes or induce stromal cells to secrete enzymes that degrade the basement membrane and matrix. For example, lysyl oxidase is an important component for the cross-linking and stabilization of the ECM. High expression of lysyl oxidase in breast CAFs induces ECM remodeling, invasion, and metastasis of breast cancer cells in vitro and in vivo. CAF-derived lysyl oxidase is an important mediator of intercellular communication in the TME and a potential therapeutic target [15, 102, 103]. Glycoprotein fibronectin is also known to be an important component of the ECM. The expression of fibronectin in primary breast tumors is closely related to tumor metastasis [104]. Breast cancer cells manipulate fibronectin matrix production by fibroblasts in a phenotypic-dependent manner [105]. Fibrillar collagen receptor disc domain receptor 2 (DDR2), which is also related to metastasis in tumors and tumor stromal cells, may also play an important role in metastasis; for example, studies have found that DDR2 is activated in CAFs, and depletion of DDR2 in CAFs leads to decreased ECM production and altered collagen structure [106]. Hao et al. used UE-SWE to monitor copy number variations (CNV) in ctDNA of breast tumors with different hardness and found that the DDR2 gene in CAFs was related to UE-SWE value and tumor hardness. After FAP was removed from CAFs by CRISPR/Cas9-mediated gene knockout, the expression of DDR2 was down-regulated, tumor hardness decreased, and the process of carcinogenesis in vivo and in vitro was inhibited [107].

In the process of CAF-induced hardening of the tumor shape to promote tumor invasiveness, mechanical signal-induced skeleton tension is an important factor in maintaining tumor stability; consequently, structural collapse is regarded as an important step in preventing or reversing the formation of CAF-induced tumors.

### The interaction between CAF and angiogenesis activates metastasis

In addition to the above micro-environment crosstalk factors, angiogenesis is another necessary step in tumor invasion and metastasis. Angiogenesis is an important condition for niche formation prior to metastasis to the TME [108]. The balance between proangiogenic and antiangiogenic factors is key to angiogenesis, in which the binding of vascular endothelial growth factor (VEGF) and its homologous receptor (VEGFR) is the classical pathway of signal transduction in tumor angiogenesis [109]. Previous studies have confirmed that mouse embryonic fibroblasts are closely associated with VEGF and promotion of angiogenesis. Although the FDA has approved anti-angiogenic drugs for VEGF or VEGFR, the clinical benefits are limited, and it has been found that the pathway independent of VEGF signaling still plays an important role in tumor progression. In breast cancer, it was found that decreased expression of miR-205 in breast fibroblasts can activate NFs to CAFs by targeting YAP1, especially without the intervention of VEGF, and CAF activation mediated by miR-205 and YAP1 can also promote angiogenesis. MiR-205/YAP1/IL11/IL15 signal axis is a VEGF-independent signal transduction pathway in breast matrix CAFs but still plays a specific role in tumor angiogenesis [43]. Similarly, high levels of Wnt5a have been detected in patients with breast cancer and are closely related to micro-vessel density in breast tumor tissues. Enhanced FOSL2 expression in CAFs is regulated by estrogen, cAMP, and PKA signal transduction. Wnt5a is a direct target of FOSL2, which promotes angiogenesis independent of VEGF in CAFs [110]. In addition to VEGF-independent angiogenesis, researchers have found that *Prunella vulgaris* polysaccharides exert anticancer effects on breast CAFs by inhibiting the expression of bFGF [111].

CAFs can affect the TME and promote tumor metastasis by secreting cytokines, remodeling the ECM, and promoting angiogenesis. For example, CAF-derived adrenomedullin plays an important role in breast cancer growth and neovascularization by providing and amplifying the signals necessary for pathological angiogenesis [112]. Similarly, interstitial fibroblasts in invasive breast cancer promote tumor growth and angiogenesis through elevated sdf-1/cxcl12 secretion [27]. LncSNHG5 is significantly up-regulated in primary breast CAFs and plays an important role in PMN formation through vascular leakage and angiogenesis [37]. As the key point in activating angiogenesis, CAFs are another important condition for the occurrence of metastasis. CAFs not only play a role in the traditional VEGF classical pathway but also play a key role in independent VEGF activation.

### Other crosstalk factors related to fibroblasts

In addition to the above factors, sex steroids are also an important part of the crosstalk between fibroblasts and TME. Specifically, steroid hormone receptors are closely related to specific cancer types. For example, androgen receptor (AR) signaling is a major driver of prostate cancer (PCa) progression [113].In contrast, estrogen is the main driving force for the regulation of recycling of the ECM in the mammary gland GPER(G protein-coupled estrogen receptor,GPER) not only mediates cell proliferation, but also promotes the adhesion / diffusion, proliferation and migration of breast CAFs [114, 115]. GPER mediates the participation of forward feedback FGF2/FGFR1 in TME, which connects CAFs with breast cancer cells and promotes tumor progression Specifically, GPER mediates the participation of forward feedback FGF2/FGFR1 in TME, which connects CAFs with breast cancer cells and promotes tumor progression [116]. Studies have found that sex steroids may affect tumor-associated stromal cells, and steroids are involved in the crosstalk between specific tumor cells and CAFs. Breast cancer-related fibroblasts and estrogen are of particular interest to us here. The effect of estrogen on the microenvironment of breast cancer is mainly reflected in the CAF-dependent induction of Tamoxifen (TAM) drug resistance, TAM and G1 induced CYP19A1 gene expression and increased E2 production, but also through the GPER/EGFR/ERK pathway [114]. As a potential therapeutic target, estrogen and fibroblasts of breast cancer provide more options for the treatment of drug resistance.

## CAFs in the diagnosis and treatment of breast cancer

The interweaving of CAFs and tumor metastasis permeates in all aspects of tumor development. It also reminds researchers that CAFs as a prognostic marker may contribute to the development of promising therapeutic drugs. In the combination therapy of targeting tumor cells, CAFs is expected to become a new strategy to improve clinical effect and overcome drug resistance. At present, aiming at the metastatic mechanism of breast cancer, the studies on the diagnosis and treatment of breast cancer by CAFs mainly include **(1)** The diagnostic and prognostic value of CAFs in BCs **(2)** The role of CAF in drug resistance **(3)** Natural products and the application of targeted CAF in combination therapy.

### The diagnostic and prognostic value of CAFs in breast cancer metastasis

CAFs infiltration and activation of CAFs-related signaling pathways are closely related to the progression and prognosis of BCs. Therefore, there are a large number of studies trying to find CAFs-related biomarkers and evaluate their value in the prognosis and early diagnosis of BC. Busch found that the loss of TGFBR2 expression in BC-related CAFs was related to tumorigenesis and metastasis. By analyzing the expression level of TGFBR2 in CAFs of 564 patients with invasive BC, it was found that the expression of CAFs-specific TGFBR2 was related to relapse-free survival [73]. In addition to directly predicting the prognosis of BCs, different properties of fibroblasts can also predict recurrence after drug treatment. CD146 positive fibroblasts can predict the improvement of relapse-free survival after tamoxifen treatment [117].

In addition, CAFs can also realize its value as an effective safeguard in predicting recurrence and metastasis when other independent prognostic factors are limited. Circulating tumor cells (CTC) are detected in both early and metastatic cancers, which makes CTC unstable as an independent prognostic factor. The addition of CAFs makes it possible to predict the metastasis and recurrence of early solid tumors. In a patient with metastatic breast cancer, CAF was detected, but the absence of CTC is a good indication of the importance of CAFs [118]. In more cases of CAFs is associated with poor prognosis, of course, more complex cases can not be excluded, such as CAFs in this case, PD-L1 can be used as a better prognostic marker for TNBC patients [119]. The chemical interaction between other stromal cells and CAF in tumor immune microenvironment can not be excluded. At this stage, there is no sufficient evidence to confirm it, and a large number of studies are needed to explore in depth [120]. Generally speaking, in evaluating the prognostic value of CAFs. CAFs is mostly associated with poor prognosis and recurrence.

### Targeted CAFs in the treatment of tumor drug resistance

In the process of clinical treatment of tumor, the problem of toxicity and side effects limits the long-term application of traditional treatment in clinical practice. Many patients are prone to drug resistance and recurrence in the late stage Broad. Researcher found that CAFs can protect breast cancer cells from the effects of adriamycin. When tumor cells were co-cultured with CAFs, the expression of interferon(IFN) in CAFs was increased, and the IFN signal pathway was activated by paracrine to induce chemotherapy resistance. IFN blocking antibody can inhibit the protective effect of CAFs on cancer cells. It is not only the paracrine of CAF that affects drug resistance, but also the paracrine of drug resistant cells can induce the activation of CAF and further drug resistance [121]. Chandra found that the expression of TGF-β in drug-resistant BC cells increased, and activated p44/42MAPK signal axis to induce CAFs activation and chemotherapy resistance by paracrine, and enhanced EMT in drug-resistant cells by inhibiting the expression of E-cadherin in CAFs [122]. Therefore, targeting TGF-β /p44/42MAPK signal pathway may help to eliminate CAFs-mediated chemoresistance. In addition to chemotherapy resistance, radiation resistance may be closely related to CAFs. Nandi found that the expression of Notch ligand Dll1 increased in patients with intracavitary BC after radiotherapy, which drives the radiation resistance and metastasis of Wnt/ β-catenin dependent Dll1+ cells. Inhibition of Dll1-mediated Notch signal can reduce the number of Dll1+ cells and CAFs, and increase the radiosensitivity of Dll1+ tumor cells [123].

### Natural products and the application of targeted CAF in combination therapy

A large number of beneficial natural compounds that can be used to counteract cancer-related inflammation or cancer progression [124] The successful treatment of cancer depends not only the destruction of the tumor cells but also the prevention of transmission of cells from the tumor medium to normal tissue, thus, preventing metastasis [125, 126]. The combined use of natural compounds and other drugs has greatly improved the efficiency of treatment. Natural compounds can be used as adjuvant chemotherapeutic drugs [127, 128]. Natural products show multitarget effects and could provide a breakthrough in the treatment of targeted metastasis in the TME [129].

The classification of the properties of natural drugs targeting breast tumor fibroblasts is not specific, and it widely involves various types of compounds, of which polyphenols and flavonoids are the main components. In addition, there are anthraquinones, alkaloids, polysaccharides and so on. This seemingly irregular attribute distribution also shows that natural medicines play a role in many ways, and do not simply rely on the division of attributes. In the process of tumor progression, the wide range of effectiveness of natural drugs also fully shows that natural products are an important therapeutic means to reverse tumor-related fibroblasts into static fibroblasts.

**Fig4:**
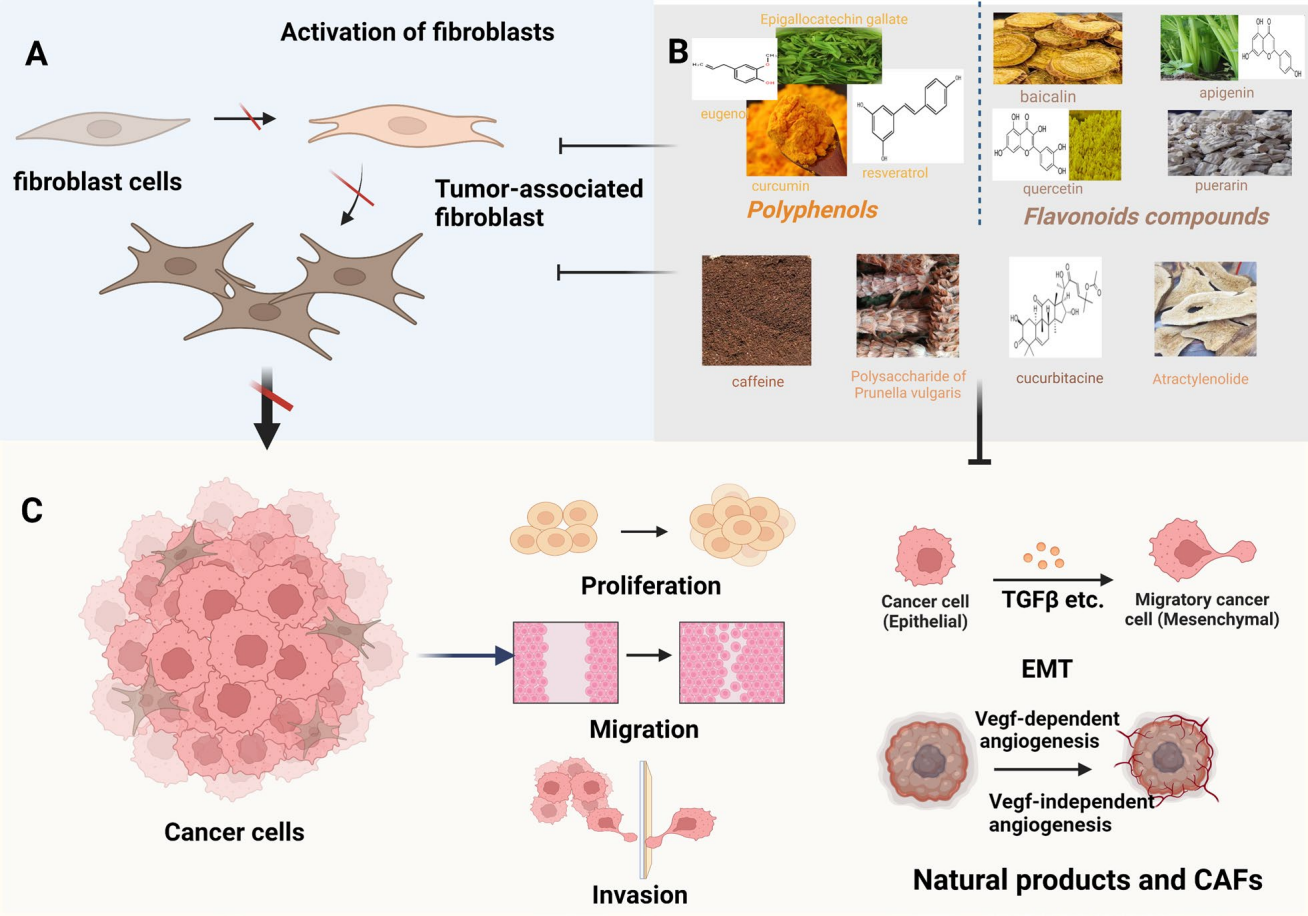
The pathway of natural products inhibiting CAF metastasis. By directly targeting CAFs and inhibiting the activation of CAFs. **A** Natural products inhibit the activation of tumor-associated fibroblasts. **B** The types of natural compounds and specific drugs that can inhibit the function of CAFs** C** The way of inhibiting tumor progression

When tumor growth and progression cannot be suppressed, natural products may be an important tool for controlling the pre-metastatic niche, as shown by the inhibition of the ability of breast active fibroblasts to induce metastatic EMT processes. Inhibition of metastasis is an important mechanism by which natural products target breast tumor-related fibroblasts, and metastasis is involved in almost all applications. Direct intervention is possible, as in the inhibitory effect of resveratrol on CAF-induced migration and invasion of breast cancer MDA-MB-231 cells [130]. Breast tumor fibroblasts cultured in different tumor areas can also be controlled by another natural product, emodin, which blocks EMT programming induced by TGF-β [131]. In addition, curcumin, caffeine, and other natural products play a similar role through the tumor-promoting paracrine role of related fibroblasts to inhibit metastasis. Another key factor associated with tumor progression and metastasis is tumor angiogenesis [132], that is, the inward growth of blood vessels, enabling the malignant cells need to enter the circulatory system and spread in the body [133]. Tumor angiogenesis is a prerequisite for tumor metastasis; the newly formed blood vessels act a bridge between the tumor and the larger circulatory system. Cucurbitacin, a STAT3 inhibitor, has been shown to inhibit the angiogenic potential of breast myofibroblasts in vivo and, by downregulating VEGF, inhibits angiogenesis in orthotopic tumor xenotransplantation [134]. In the current study, in addition to traditional VEGF-dependent angiogenesis, there was also non-VEGF-dependent angiogenesis, which may be achieved through paracrine signaling of CAF cells and may act on other angiogenic effectors. For example, researchers have found that paracrine-mediated *Prunella vulgaris* polysaccharides can exert anticancer effects on breast CAFs by inhibiting the expression and biological activity of bFGF [111]. There are, however, few meaningful studies in this field, to some extent, this reflects that VEGF-independent angiogenesis could offer another key breakthrough in the study of tumor-related fibroblasts that induce angiogenesis. Here we use the table to summarize the treatment of CAFs in breast cancer, in which the inhibition of breast CAFs by natural products attracts our attention. Table 3 and Fig. 4 show the mode of action and pathways of natural products related to breast cancer fibrogenesis.

**Table 3:** Natural compounds targeting brast CAF

Natural product	Species	Mechanism	Origin and/or function	Refs.
Resveratrol	Polyphenols	resveratrol inhibits CAF-CM-induced breast cancer cell migration and invasion by blocking cyclin D1 and c-Myc expression	Migration and invasion of breast cancer	[130]
Curcumin	Polyphenols	Reduces the secretion of IL-6, MMP2, MMP9, transforming growth factor-β and SDF-1; Upregulates the aging-related proteins P16, P21 and P53	Inhibits the invasion/migration of breast cancer cells and induces fibroblast aging	[135]
ECGG	Polyphenols	Reduce HGF in CAFs to inhibit tumor proliferation and invasion, and inhibit tumor metastasis by lowering VEGF	Proliferative, migratory and invasive tumors	[136]
Eugenol	Polyphenols	Eugenol downregulates DNMT1 and DNMT3A by inhibiting the migratory/invasion capacity of CAFs by inhibiting Erk1/2 and MMP-9	Migration, invasion and EMT of breast cancer	[137]
Eugenol	Polyphenols	Eugenol and decitabine inhibit paracrine proangiogenic effects of CAF cells by inhibiting DNMT1 and its downstream angiogenic effectors VEGF-A and IL-8	angiogenesis	[138]
Oroxylin A	Flavonoid	OA can inhibit phosphorylation of FAK/STAT3 by reducing the expression of ACTN1	Proliferative, migratory and invasive tumors	[139]
Puerarin	Flavonoid	Reduces intratumoral immunosuppressive cytokines (IL-4, IL-6, IL-10 and IL-13)	Downregulate CAFs to increase infiltrating T cells in tumors	[140]
Apigenin	Flavonoid	Apigenin can block the release of CCL2 in TNFα-mediated triple-negative breast cancer (TNBC) cell lines	Blocks the release of chemokines	[141]
Caffeine	Alkaloid	Caffeine inactivates CAFs cells via PTEN-dependent Erk1/2 and Akt inactivation	Migration, invasion and angiogenesisof breast cancer	[142]
Prunella Vulgaris Polysaccharide	PolysacchArides	PVP can exert anticancer effects on breast CAFs by inhibiting the expression of bFGF	Inhibits tumor growth, migration and angiogenesis	[111]
Cucurbitacine	Triterpenoid	Acts on breast CAFs by IL-6/STAT3/NF‐κB	Inhibits paracrine and angiogenesis	[134]
Atractylenolide-I	Lipid components	ATL-1 downregulates fibroblast expression of CTGF	Inhibits fibroblast transformation ability and increases chemotherapy sensitivity	[143]

The bioavailability of many natural compounds is usually far from satisfactory, and this limits the efficacy of natural drugs to some extent. Accordingly, nanocarriers are widely used by researchers. Nanotechnology helps to improve targeting efficiency, and CAF is the main participant in limiting drug penetration. Elimination of CAF may reduce the content of collagen in ECM and improve the accumulation and penetration of nanoparticles in tumors [98, 144]. Fibroblast combined with chemotherapeutic drug delivery nanoparticles may be a promising combined therapy strategy for breast cancer, and its efficacy is mainly focused on two aspects: on the one hand, nanoparticles can effectively inhibit the formation of tumor-related fibroblasts [145, 146]. On the other hand, it can increase the therapeutic effect, reduce toxicity and increase efficiency, and activate the immune microenvironment. Zhang constructed a graded bioresponsive nanoparticles (R (D) / H (S) NPs). Doxorubicin (DOX) and TGF-β receptor inhibitor (SB431542) were loaded on the nanoparticles. Nanoparticles can effectively inhibit the formation of CAFs, activate the immune microenvironment, enhance the chemotherapeutic effect of doxorubicin and inhibit breast cancer metastasis [147]. Secondly, glycolipid-based polymer micelles (GLPM) encapsulated angiotensin II receptor I inhibitors and cytotoxic drugs (doxorubicin, DOX) pre-administered GLPM/Tel fully reduced the matrix components derived from CAF, and constructed the basic for accurately reaching the location of the disease, whether it is bioresponsive nanoparticles or drug delivery. On the basis of inhibiting CAF, the efficacy of chemotherapeutic drugs was improved and the toxicity and side effects were reduced [148].

At present, the application of natural products in anti-breast tumor fibroblasts is mainly concentrated in three different stages. First of all, kill the growth of the tumor at the “source”. Secondly, when the body is unable to suppress the growth and progress of the tumor, natural products may be an important weapon to control the niche before metastasis. it is shown by inhibiting the ability of breast cancer cells to promote metastasis and inhibiting angiogenesis. Finally, in the field of application, the combination of natural products with nanomaterials and chemotherapeutic drugs fully avoids the problems of low oral utilization and drug absorption of natural products, and gives full play to the characteristics of natural products in the treatment of breast tumors.

## Opportunities and challenges in targeting breast cancer fibroblasts to arrest the progress of metastasis

The metastasis and recurrence of breast cancer are owing to not only the existence of invasive tumor cells but also the appropriate microenvironment, which is an important driving factor of metastasis [149]. The cascade reaction of metastasis largely depends on the ability of cancer cells to adapt to distant tissues and corresponding new microenvironments, in which interstitial cells and various cytokines, extracellular vesicles, ECM, and immune cells are the “accomplices” in tumor progression [150]. At present, the ecotherapy strategy of reversing and reshaping the TME is mainly focused on cutting off the relationship between different elements in the tumor. In this review we have summarized the complex interactions between breast CAFs and various components of the TME during the progression of metastasis.

It is necessary to block communication between tumor cells and fibroblasts. Both soluble cytokines and exocrine bodies act as transmitters that promote the metastasis cascade. It has been confirmed that not only is the influence between tumors and fibroblasts not in a single direction but the factors secreted by fibroblasts and exocrine bodies can also aggravate the progression of tumors. Cutting off the communication between the tumor and fibroblasts is an important strategy for breaking this vicious circle. At present, efforts to develop this solution have mainly focused on the relevant signal axis, such as growth factor-related TGF-β 1/Smad or chemokine-related CXCL1/CXCR12 signal axis. The crosstalk between fibroblasts and immune cells is mainly concentrated on macrophages in innate immune cells. Between CAFs, M2 polarized macrophages, and tumors, the spread and metastasis of cancer cells are realized through cross-interactions between stromal cells, cancer cells, and secreted cytokines. In the process of targeting CAFs, ECM acts as a barrier to protect cancer from treatment and support tumor progression. Destruction of the ECM is the defense layer that destroys TME, so targeting ECM is a potential strategy for anti-malignant therapy [151]. In addition, fibroblasts cannot be ignored in the field of anti-angiogenesis and unique estrogenic effects. Among the studies on estrogen-related breast cancer subtypes, CAF is more focused on hormone resistance research.

Natural products play an important role in preventing fibroblasts from promoting tumor cell transformation. Comprehensive studies on natural products in tumor-related fibroblasts have focused on reversing tumor metastasis. It is undeniable that at present, the combination of natural products and other chemotherapeutic drugs to target CAF is a very potential development direction, which not only makes up for the shortcomings of natural products, but also gives full play to its advantages [128].

Although targeted CAFs have great application prospects and therapeutic potential, the heterogeneity of breast cancer fibroblasts requires further study. In the case of breast cancer, there was no significant relationship between the expression of CAF-related proteins and molecular subtypes. By contrast, CAFs were found to be correlated with metastatic organs [152]. Secondly, understanding the regulatory relationship between activated fibroblasts and the adaptive immune system will greatly promote the study of the tumor immune microenvironment. Although research in this area is limited, the limitations suggest that CAFs are an effective target for solving the metastatic cascade effect. The analysis of the highly complex interaction between CAFs and tumors is a viable approach to inhibiting tumor progression, and the targeted therapy of CAFs has unlimited possibilities.

## Data Availability

Not applicable.

## Abbreviations

CAFs: Cancer-associated fibroblasts
TME: Tumour microenvironment
ECM: Extracellular matrix
IL: Interleukin
MSCs: Mesenchymal stem cells
EMT: Epithelial–mesenchymal transition
DDR2: Disc domain receptor 2
CNV: Copy number variations
TGF-β: Transforming growth factor-β
HGF: Hepatocyte growth factor
PDGF: Platelet derived growth factor
OPN: Osteopontin
IL-6: Interleukin-6
IL-6R: IL-6 receptor
TNBC: Triple-negative breast cancer
TAMs: Tumor-associated macrophages
TANs: Tumor-associated neutrophils
VEGF: Vascular endothelial growth factor
VEGFR: Vascular endothelial growth factor Receptor
NFs: Normal fibroblasts
CTC: Circulating tumor cells
IFN: Interferon
BFGF: Basic fibroblast growth factor
R (D)/H (S) NPs: Graded bioresponsive nanoparticles
DOX: Doxorubicin
GLPM: Glycolipid-based polymer micelles
GLIS1: Targeting Gli-similar1
TNF-α: Tumor necrosis factor-α
BMP: Bone morphogenetic protein
PMN: Premetastatic niche
